# Cardioprotective Effect of Danhong Injection against Myocardial Infarction in Rats Is Critically Contributed by MicroRNAs

**DOI:** 10.1155/2019/4538985

**Published:** 2019-08-26

**Authors:** Jingrui Chen, Jing wei, John Orgah, Yan Zhu, Jingyu Ni, Lingyan Li, Han Zhang, Xiumei Gao, Guanwei Fan

**Affiliations:** ^1^First Teaching Hospital of Tianjin University of Traditional Chinese Medicine, Tianjin 300381, China; ^2^Tianjin State Key Laboratory of Modern Chinese Medicine, Tianjin University of Traditional Chinese Medicine, Tianjin 301617, China; ^3^Ministry of Education, Key Laboratory of Formula of Traditional Chinese Medicine, Tianjin 301617, China; ^4^Tianjin Key Laboratory of Traditional Chinese Medicine Pharmacology, Tianjin University of Traditional Chinese Medicine, Tianjin 301617, China

## Abstract

**Background:**

Danhong injection (DHI) has been mainly used for the treatment of myocardial infarction, atherosclerosis, and coronary heart disease in clinical practice. Our previous studies have shown that DHI improves ventricular remodeling and preserves cardiac function in rats with myocardial infarction (MI). In this study, we focused on the potential mechanism of DHI in protecting cardiac function in MI rats.

**Methods:**

Sprague-Dawley rats were subjected to ligation of the left anterior descending coronary artery (LAD) to prepare a myocardial infarction (MI) model. After 14 day DHI intervention, cardiac function was measured by echocardiography and myocardial fibrosis was assessed by Masson staining. Differentiated miRNAs were screened using rat immunopathology miScript miRNA PCR arrays, and their results were verified by RT-PCR, immunofluorescence, and immunoblotting.

**Results:**

DHI treatment significantly reduced infarct size and improved cardiac function and hemodynamics in MI rats by echocardiography and morphology. miRNA PCR array results showed that DHI reversed 25 miRNAs known to be associated with inflammation and apoptosis. Moreover, the expression of inflammatory factors TNF-*α*, IL-1*β*, and IL-6 was significantly reduced in the treated DHI group. Mechanistically, DHI downregulated the inflammatory transcription factor NF-*κ*B (as reflected by inhibition of NF-*κ*B p65 nuclear translocation and phosphorylation of the I*κ*B*α*).

**Conclusions:**

DHI is effective in mitigating inflammation associated with MI by preventing NF-*κ*B nuclear translocation and regulating miRNAs, thereby improving cardiac function in myocardial infarction rats.

## 1. Introduction

Acute myocardial infarction (AMI) is a common cardiovascular disease with a high mortality rate [1]. Important medical and interventional therapies have been incorporated into the clinical guidelines and shown to reduce the incidence and mortality of acute myocardial infarction in randomized clinical trials [2]. Inflammation is frequently encountered in clinical practice, and various causes can cause myocardial inflammation and lead to defects in the myocardial structure and function [3, 4]. The inflammatory response is a prerequisite for scar formation and healing, which can reduce deleterious myocardial remodeling by promoting effective tissue repair after MI [5]. However, overproduction of inflammatory mediators and proinflammatory cytokines can lead to the deterioration of the pathological process. Inhibition of excessive inflammation may allow us to design effective inﬂammatory-related interventions for treating MI.

MicroRNAs (miRNAs) are endogenous, conserved, single-stranded, small (approximately 22 nucleotides), noncoding RNAs expressed in a cell and tissue-specific manner that regulate gene expression and organ function, including the myocardium [6]. miRNA plays a mediating role in gene expression by combining the 3′-UTR of target genes at the posttranscriptional level by either promoting degradation or inhibiting translation—repress gene expression [7]. Each miRNA can regulate several to hundreds of different target genes by inducing degradation of its target mRNA and inhibiting translation [8, 9]. It is widely recognized that miRNAs represent critical regulators of cardiovascular function and show potential as circulating biomarkers of cardiovascular diseases since the discovery of their presence and stability in peripheral blood [10, 11], Several studies reported miRNAs have a crucial role in the pathogenesis and progression of heart failure and a huge diagnostic potential in the setting of AMI and its ability to downregulate speciﬁc genomic network expressions [12–16]. Increasing evidence shows that miRNAs play functional roles in the development of heart failure and a dysregulation of miRNA expression in the failing or hypertrophic heart [14, 16–20]. Recent research reveals a crucial role of miRNAs as a mediator in the inflammation cascade [21].

More effective and safe anti-inflammatory agents are being investigated, including nonsteroidal anti-inflammatories and traditional Chinese medicine (TCM), with many more compounds under development, such as Xuebijing injection and Shenfu injection have been used for treating inﬂammatory response, and both of them have resulted in satisfactory effects [22–24]. Danhong injection (DHI) is a standard extract from danshen (Salvia miltiorrhiza Bunge, Labiatae) and honghua (Carthamus tinctorius L, Compositae), which has been long used primarily for the treatment of ischemic encephalopathy and cardiac diseases including MI and angina in clinics [25, 26]. Our previous study showed that the main components of DHI contain the following substances: danshensu, hydroxysafﬂor yellow A, 5-hydroxymethyl-2-furfural, protocatechuic aldehyde, lithospermic acid, caffeic acid, salvianolic acid A, salvianolic acid B, salvianolic acid C, protocatechuic acid, and rosmarinic acid [27]. And research has showed that DHI prevented the lipopolysaccharide-stimulated systemic acute inﬂammatory reaction via inhibiting the expressions of iNOS, COX-2, IL-1*β*, IL-6, MCP-1, and TNF-*α* [28]. This result suggests that DHI may act on several targets to alleviate inflammation.

We recently reported that DHI could be used for ventricular remodeling after MI [29]. In this present study, we mainly investigated whether DHI could delay ventricular remodeling and protect cardiac function by inhibiting inflammation in the MI model and its mechanism of action.

## 2. Materials and Methods

### 2.1. Drugs and Reagents

DHI (Country Medicine Accurate Character Number: Z20026866, Batch number: 13062020) was obtained from Heze Buchang Pharmaceutical Co., Ltd. China. Valsartan (Batch number: X1651) was obtained from Beijing Novartis Pharma Co., Ltd. China. Chloral hydrate (Batch number: Q/12HB 4218-2009) was purchased from Tianjin Kermel Chemical Reagent Co., Ltd. China.

The Bcl-2 (PAA778Ra81) and caspase-3 (PAA626Ra81) primary antibodies were purchased from Uscn Life Science Inc. (Wuhan, China). The *α*-sarcomeric actin primary antibodies were purchased from Sigma Chemical Company (USA). Primary antibodies anti-phospho-p65 (ab86299), anti-phospho-I*κ*B-*α* (ab12135), anti-I*κ*B-*α* (ab109300), and anti-Lamin B1 (ab133741) were purchased from Abcam Inc. (Cambridge, UK). The anti-p65 (CST3034s) and anti-GAPDH (CST5174) primary antibodies and the secondary antibodies were obtained from Cell Signaling Technology (Danvers, MA, USA). The tumor necrosis factor-alpha (TNF-*α*), interleukin-1*β* (IL-1*β*), and interleukin-6 (IL-6) assay kit were from Uscn Life Science Inc. (Wuhan, China).

### 2.2. Quality Control of DHI

According to the National Drugs surveillance administrative bureau standard WS-11220, the total amount of danshensu (molecular formula: C_9_H_10_O_5_) and protocatechuic aldehyde (molecular formula: C_7_H_6_O_3_) should not be lower than 0.5 mg in 1 mL DHI analyzed by high-performance liquid chromatography (HPLC) as a reference in 1 mL injection. Simultaneously, the content of the total flavonoids determined by visible light spectrophotometry should not be lower than 5.0 mg/mL against rutin (molecular formula: C_27_H_30_O_16_) as a reference in 1 ml injection [30].

As early as 2012, our laboratory has established a dual-standard quality assessment of DHI [27]. HPLC ﬁngerprinting of DHI has been performed on the ACQUITY UHPLC BEH C18 column (2.1 × 150 mm^2^, 1.7 m) and an C18 guard column. The quality control of the DHI method is as follows. The flow rate was 0.3 mL/min. The column temperature was 30°C. The mobile phase comprised (A) aqueous phosphoric acid (0.2%, v/v) and (B) acetonitrile using a gradient elution of 5–5% B at 0–3 min, 20–20% B at 15–20 min, 27–27% B at 25–28 min, and 27–70% B at 28–30 min, and the reequilibration time of gradient elution was 2 min. Moreover, the detection wavelength was set at 280 nm [31].

### 2.3. Animals

Male Sprague-Dawley rats, weighing 220–250 g, was purchased from Beijing HFK Bioscience. Co., Ltd. The rats were raised at 12 h dark/light cycle with a temperature of 22 ± 2°C and humidity of 40 ± 5%. Standard rat chow and clean drinking water were provided ad libitum.

The study was carried out in accordance with the recommendations in the guidance for the Care and Use of Laboratory Animals issued by the Ministry of Science and Technology, China. All animal experiments and procedures were approved by Tianjin University of Traditional Chinese Medicine (TCM) Laboratory Animal Ethical Committee (TCM-LAEC2017026), and all procedures were performed to reduce the number and animals and suffering during the experiments.

Rats were anesthetized with chloral hydrate (5%, peritoneal injection, 300 mg/kg). Adequacy of anesthesia was controlled by monitoring corneal reﬂex and the lack of response to toe-pinching. Euthanasia was performed by excessive inhalation of isoflurane. Death was monitored by the cardiac activity and respiration.

### 2.4. Inducing Myocardial Infarction in a Rat and Drug Administration

For the MI rat model, the left anterior descending (LAD) coronary artery was subjected to ligation according to the previous studies [32], and the specific process was carried out as previously reported [29].

Rats were randomly divided into four groups (*n* = 10/group): (1) sham (normal saline, intramuscular); (2) model (normal saline, intramuscular); (3) DHI (DHI, intramuscular); (4) valsartan (intragastric). The clinically used dosage of DHI and valsartan was converted for use in our experiment 0.76 ml/kg/day and 10 mg/kg/day, respectively. The substances were routinely administration for 14 days.

### 2.5. Echocardiographic and Hemodynamic Assessment of Left Ventricular Function

The functionality of the left ventricle was evaluated at 14 days after MI, using a Vevo 2100 Ultrahigh resolution small animal ultrasound imaging system in real time (VisualSonicsVevo 2100, Canada), as previously described [33]. The cannulation was done to the left ventricle through the right carotid artery, which was connected to a biofunction experiment system MP100-CE (BIOPAC systems Inc., Santa Barbara, California, USA) at the end of the echocardiography experiment. The detailed procedure is described in our earlier report [29].

### 2.6. MicroRNA PCR Array

The total RNA was extracted from the LV samples (Infarct border zone, 30 mg/each rat) with miRNeasy Mini kit (Qiagen, Germany) according to the manufacturer's instructions. The RNA samples (2 *μ*g) were reverse transcribed to cDNA with the miScript II RT kit (Qiagen, German). The reaction system and conditions are shown in Table 1.

Using miScript PCR Starter Mix Reagent kits (Qiagen) and miScript miRNA PCR arrays (rat immunopathology miRNA PCR array: MIRN-104Z) to run the Q-PCRs, miRNAs expression was confirmed by specific primers. The reaction system and cycling conditions are shown in Tables 2 and 3.

### 2.7. Quantitative Real-Time PCR

Real-time PCR in a StepOne Real-Time PCR system (Applied Biosystems) was used to determine the cardiac mRNA expression of IL-1*β*, TNF-*α*, caspase-3, Bcl-2, and GAPDH. The sequences of the sense and antisense primers used for amplification are shown in Table 4.

### 2.8. Serum Biochemical Indices

At the 7th and 14th day after surgery, tagged as the inflammatory reaction period, serum was also collected from the rats. The levels of inflammatory factors which involve TNF-*α*, IL-1*β*, and IL-6 were measured by commercially available ELISA kits according to the manufacturer's instructions.

### 2.9. Western Blot Analysis

Myocardial tissues were cut from the infarct border zone. Cytosolic and nuclear proteins were extracted from the infarct border zone of the heart tissue using NE-PER nuclear and cytoplasmic extraction reagents according to the manufacturer's instructions (Thermo Scientiﬁc, USA). Proteins were separated by SDS-PAGE (8–10%) and probed with different primary antibodies against p-P65, NF-*κ*B, p-I*κ*B-*α*, GAPDH, and Lamin B.

### 2.10. Histopathological Examination

Myocyte size and interstitial fibrosis were shown by pathological section. Pathological examination was performed using the paraffin sections and stained with hematoxylin and eosin (H&E) and Masson trichrome. Infarct size measurements were performed by measuring the midline length [34].

### 2.11. Immunofluorescence

Immunofluorescence was performed with standard protocols on the basis of H&E staining. Cardiomyocytes were marked with sarcomeric actin (A2172, Sigma), while DAPI (70317525, Roche) was used as the nucleus marker. Apoptosis was determined by expression of cleaved caspase-3 (PAA626Ra81, USCN) and Bcl-2 (PAA778Ra81, USCN). Fluorescence images were captured by use of OLYMPUS DP71 inverted fluorescence microscopy.

### 2.12. Statistical Analysis

All data in the experiment were expressed as mean ± SD. Data analyses were performed using the SPSS 17.0 software package. Statistical analysis was assessed by the analysis of variance (ANOVA) followed by the LSD test or Dunnett's *t*-test for multiple comparisons. The differences were considered statistically signiﬁcant at the value of *p* < 0.05.

## 3. Results

### 3.1. Treatment with DHI Improves Cardiac Performance and Hemodynamics in the MI Rat Model

**Figure 1:**
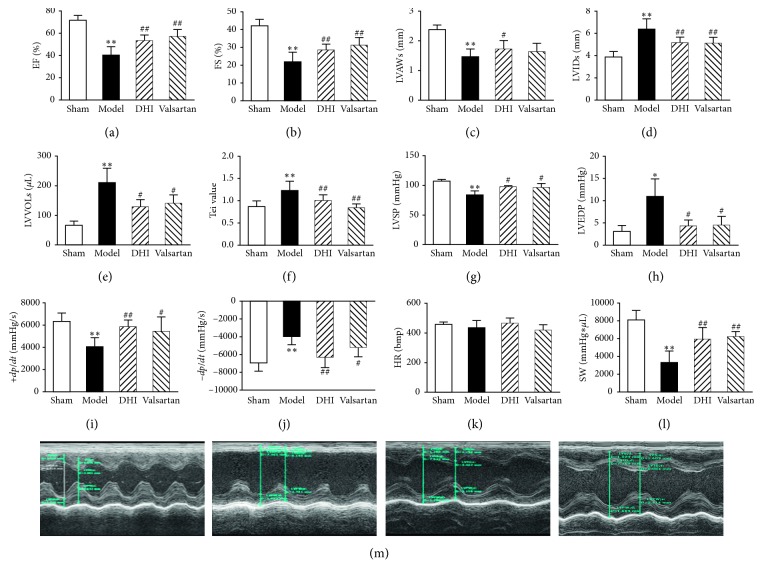
Effects of DHI on cardiac functionality and hemodynamics index. Quantitative assessment of dilation and systolic function based on LVEF (LV ejection fraction) (a), LVIDs (LV end-systolic dimensions) (b), LVAWs (LV end-systolic anterior walls) (c), LVFS (LV fractional shortening) (d), LVVols (LV systolic volumes) (e), Tei value ((IVCT + ICRT)/MVET) (f), isovolumic contraction time plus isovolumic relaxation time (IVCT + IVRT), mitral valve ejection time (MVET), and LVSP (LV systolic pressure) (g), LVEDP (LV end-diastolic pressure) (h), +*dp*/*dt* max (LV maximum upstroke velocity) (i), −*dp*/*dt* max (LV maximum descent velocity) (j), HR (heart rate) (k), and SW (stroke work) (l). Representative echocardiographic images (M mode) in different groups (m). From left to right: sham group, model group, DHI group, valsartan group. All values are means ± SD (*n* = 8 or *n* = 6). ^*∗*^*p* < 0.05 and ^*∗∗*^*p* < 0.01 versus sham group; ^#^*p* < 0.05 and ^##^*p* < 0.01 versus model group.

Cardiac function was measured 14 days afterward via echocardiographic assessments (LVEF, LVFS, LVAWs, LVIDs, LVVOLs, and Tei value). LVEF, LVFS, and LVAWs were significantly greater in rats administered with DHI and valsartan than in those treated with saline, while LVIDs, LVVOLs, and Tei value were significantly smaller (Figures 1(a)–1(f)). The representative echocardiograms in different groups are presented in Figure 1(m). Hemodynamic parameters measured via an intracardiac Millar catheter are presented in Figures 1(g)–1(j). Rats with MI developed systolic dysfunction, as evidenced by significantly decreased LVSP and the maximum rate of rising in LV pressure (+*dp*/*dt* max), also exhibited a severe diastolic dysfunction, as defined by elevated LVEDP and the maximum rate of decline in LV pressure (−*dp*/*dt* max) in the model group than the sham group. DHI and valsartan administration significantly improved systolic cardiac function, enhancing LVSP and +*dp*/*dt* max, and provided beneficial effects on the diastolic function by reducing LVEDP and −*dp*/*dt* max compared to saline-treated rats (*p* < 0.05). DHI significantly ameliorates cardiac function, by increasing SW (5951.33 ± 1313.81 compared to the model 3307.83 ± 1304.61 mmHg*∗μ*L; *p* < 0.01), whereas HR did not differ significantly between all groups at 2 weeks after MI (Figures 1(k) and 1(l)).

### 3.2. Myocardium Histology

**Figure 2:**
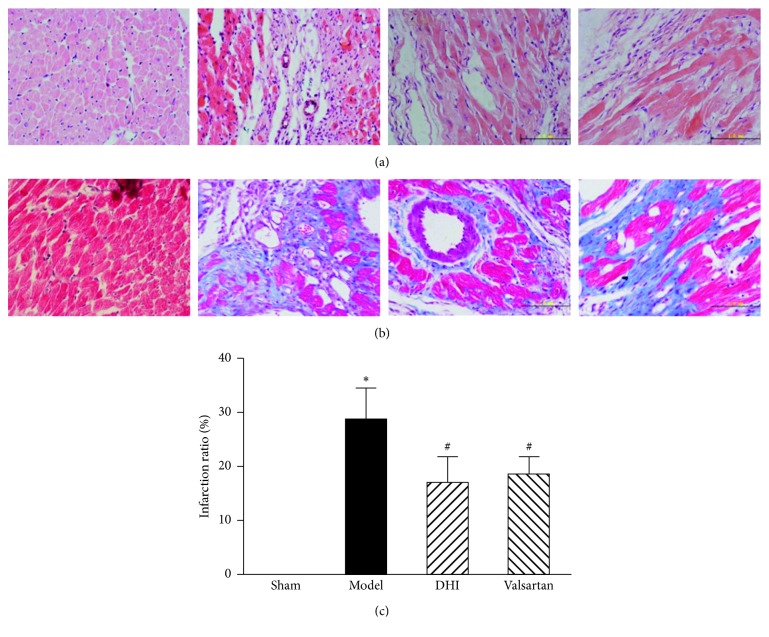
Left ventricle stained with H&E and Masson. (a) Representative photomicrographs of H&E-stained myocardium (400×). (b) Representative photomicrographs of Masson-stained myocardium (400×). From left to right: sham group, model group, DHI group, and valsartan group. (c) Proportion of the infarct ratio represented in bar graph; the cavity on the right represents left ventricular; red represents cardiomyocytes, and blue represents collagen fiber. All values are expressed in means ± SD, (*n* = 5). ^*∗*^*p* < 0.05 versus the sham group; ^#^*p* < 0.05 versus the model group.

We directly observed the effect of DHI on the myocardial structure and gross morphology after myocardial infarction by H&E and Masson staining (Figure 2). Morphological observation of H&E staining showed increase in myocardial hypertrophy and cell gap, loose of the structural arrangement of myocardial cells, rupture of myocardial ﬁbers, and inflammatory cell infiltration, while a relative slighter condition in drug-treated groups was observed (Figure 2(a)). Less myocardial fibrosis is found in the heart after DHI and valsartan treatment as demonstrated by Masson staining (Figure 2(b)). Interstitial collagen density was markedly increased in the LV myocardium of the rat model than the sham group. Treatment with DHI significantly reversed this effect. The statistical infarction ratio was conducted by the application of the midline method. Compared with the model group, ratio of infarct was markedly decreased in the DHI and valsartan treatment group (Figure 2(c)).

### 3.3. MircoRNA Expression Pattern in DHI-Treated MI Rats Model

The rat immunopathology miScript miRNA PCR array was applied to screen for differential miRNA, and the miRNA PCR array result revealed that there were 5 upregulated and 15 downregulated miRNAs genes (fold change > 2) in the model group compared to the sham group and 25 upregulated miRNAs genes (fold change > 2) in the DHI-treated group compared to the model group. These significant differences in miRNAs are related to the immune response and inflammation, as shown in Tables 5 and 6.

### 3.4. Role of miRNAs in the MI Rat Model as Revealed in Microarray-Based Bioinformatics Prediction

**Figure 3:**
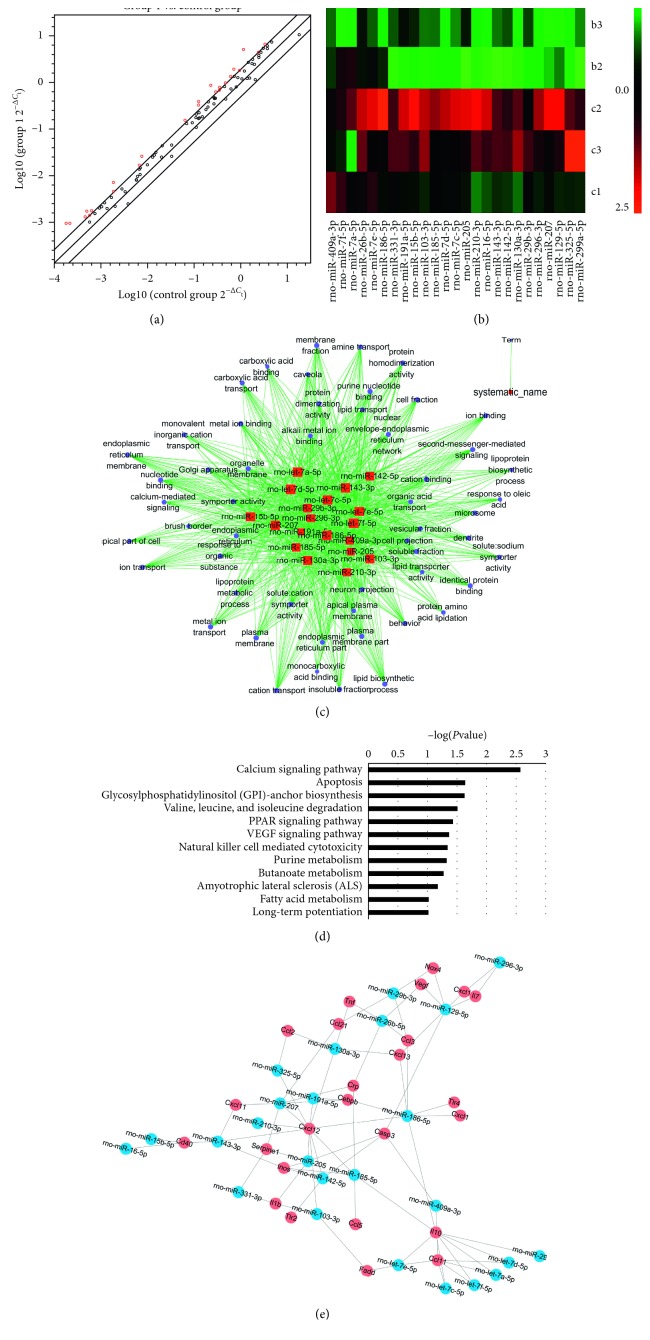
Regulation of DHI on miRNA through the immunopathology miScript miRNA PCR array analysis. (a) Scatter diagram of differentially expressed miRNA of the DHI group upon model group. 2-fold regulation was selected, with DHI as group 1, and MI model was selected as the control group. Red indicates upregulated genes. miRNA above the oblique line is upregulated and below the line is downregulated (*n* = 3). (b) Unsupervised hierarchical clustering analysis on the differentially expressed miRNAs (>2-fold change) in the model group vs DHI group. Note: (1) red represents upregulation of miRNA; green represents downregulation of miRNA. (2) (b) model group and (c) DHI group. (c) miRNA-GO-network was generated according to the relationship of significant functions and miRNAs. A high enrichment degree between the differentially expressed miRNAs and the functions in the network. Enrichment degree means the contribution of a miRNA to the surrounding GOs or the opposite. The blue circle represented GOs, the red square represented miRNAs, and their relationship was represented by lines art. (d) The miRNA-KEGG signal pathway analysis of the differentially expressed miRNA related to the signal pathway. The black bar represents the signal pathway regulated by overexpressed and underexpressed miRNAs. The vertical axis is the pathway category, and the horizontal axis is the −log *p* value of each pathway. (e) Using differentially expressed miRNA and prediction target gene to construct the miRNA-mRNA network. The blue circles represent miRNAs, the red circles represent mRNA, and lines art represent the relationship between miRNA and mRNA.

In order to obtain a better overview of miRNA expression signature, we further performed scatter diagram and unsupervised hierarchical clustering heat map analysis of differentially expressed miRNA by normalized probe signal values (Figures 3(a) and 3(b)). First and foremost, the miRNA PCR array was used to analyze miRNA expression profiles in the model and DHI group. Fold change > 2 and *p* value < 0.05 between model and DHI group were set as the criteria for filtering differently expressed miRNAs. The scatter diagram result revealed that the expression of 25 miRNAs was strongly upregulated under 14-day DHI treatment. The heat map showed differentially expressed miRNA profiling.

According to the selected differentially expressed miRNA (fold change > 2), we have carried out the target gene prediction through the GeneSpring and microRNAorg database. The *p* value of the predicted target gene was calculated by hypergeometric distribution. GO and KEGG analysis was performed on the target gene with *p* ≤ 0.05 using David software, and a miRNA-mRNA network was constructed.

In order to predict which possible functions and biological pathways were affected in the MI rat model and DHI group, we performed gene ontology (GO) and KEGG pathways analysis of 19 miRNAs which significantly changed after pretreatment with DHI. Bioinformatics analysis predicted that 19 miRNAs up- or downregulated 54 GOs significantly (refer miRNA-GO-network (Figure 3(c)) for more detailed information). Moreover, miRNA-KEGG signal pathway analysis revealed that the molecular pathways regulated by the differential miRNAs in the expression level mainly included the calcium signaling pathway, apoptosis, glycosylphosphatidylinositol-anchor biosynthesis, valine, leucine, and isoleucine degradation, PPAR signaling pathway, VEGF signaling pathway, natural killer cell-mediated cytotoxicity, purine metabolism, butanoate metabolism, amyotrophic lateral sclerosis, fatty acid metabolism, and long-term potentiation (Figure 3(d)).

The miRNA-mRNA network was constructed based on the relationship between differentially expressed miRNAs and their target genes. The results show that the differential miRNAs such as let-7a-5p, miR-103-3p, miR-142-5p, miR-143-3p, miR-185-5p, miR-186-5p, miR-26b-5p, miR-205, and miR-207 constituted intensive molecular networks and participated in the regulation of the immune response, e.g., TNF-*α*, IL-1*β*, and caspase-3 (Figure 3(e)), Table 6). This result poses an important question which led to the further experiment.

### 3.5. Effect of DHI on Prediction of Target Gene Expression

**Figure 4:**
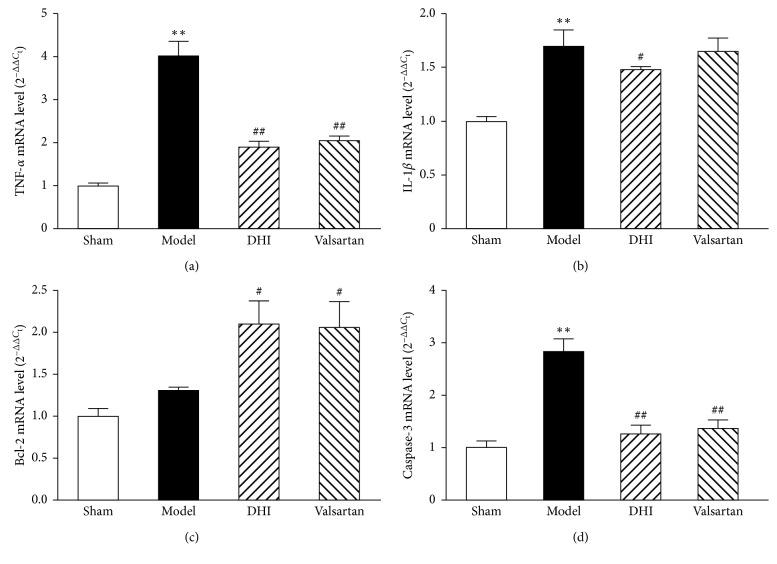
Effects of DHI on mRNA expression in myocardial tissue following MI. The relative levels of cardiac TNF-*α*, IL-1*β*, Bcl-2, and caspase-3 mRNA were assessed by RT-PCR. Results were normalized to GAPDH, and all values are expressed in means ± SD (*n* = 4). ^*∗*^*p* < 0.05 and ^*∗∗*^*p* < 0.01 compared with the sham group; ^#^*p* < 0.05 and ^##^*p* < 0.01 compared with the model group.

Based on miRNA PCR array results, hearts taken from rats in each of the experimental group were used for the confirmation of TNF-*α*, IL-1*β*, caspase-3, and Bcl-2 on the mRNA level (Figure 4). The RT-PCR result showed that mRNA expressions of TNF-*α*, IL-1*β*, and caspase-3 were markedly upregulated in the model group (*p* < 0.01, compared to the sham group). Interestingly, treatment with DHI and valsartan signiﬁcantly downregulates mRNA expression of TNF-*α* and caspase-3 (*p* < 0.01), whereas DHI alone markedly reduced IL-1*β* mRNA expression (*p* < 0.05) in the MI rat model. For the mRNA expression of Bcl-2, DHI and valsartan increased Bcl-2 mRNA expression notably (*p* < 0.05 and *p* < 0.01).

### 3.6. Effects of DHI on the Serum Level of Inflammatory Cytokines

**Figure 5:**
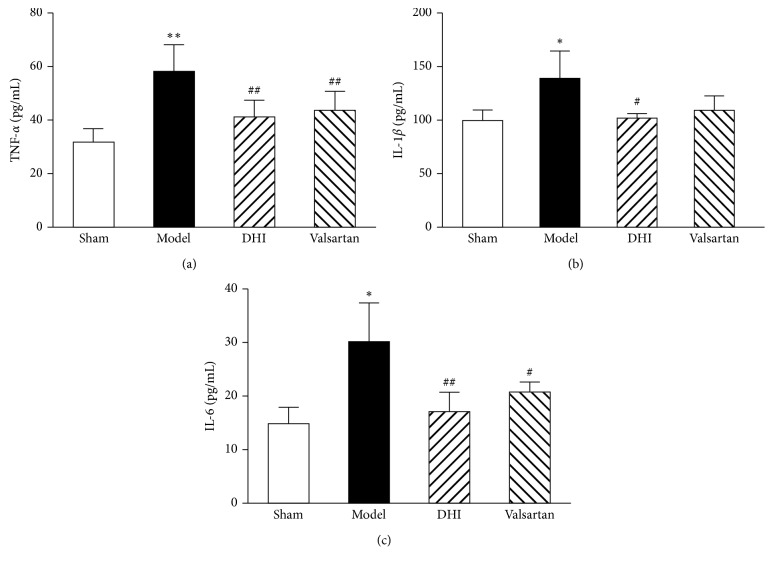
Inhibitory effect of DHI on inflammatory cytokines. (a–c) Effects of DHI on serum TNF-*α*, IL-1*β*, and IL-6 levels recorded at the end of 14 days after surgery; all values are expressed in means ± SD (*n* = 8). ^*∗*^*p* < 0.05 and ^*∗∗*^*p* < 0.01 compared with the sham group; ^#^*p* < 0.05 and ^##^*p* < 0.01 compared with the model group.

To further validate the prediction results, TNF-*α*, IL-1*β*, and IL-6 were measured in the serum at 14 days postsurgery. A shown in the results displayed in Figure 5, rats with MI had a higher serum level of TNF-*α*, IL-1*β*, and IL-6 concentration than that in the sham group (*p* < 0.05). These cytokines levels in the DHI group were significantly decreased (*p* < 0.05 and *p* < 0.01), while the valsartan group showed the same effect, but there was no significant difference in IL-1*β*.

### 3.7. DHI Modulates NF-*κ*B Signaling Pathway in the MI Rat Model

**Figure 6:**
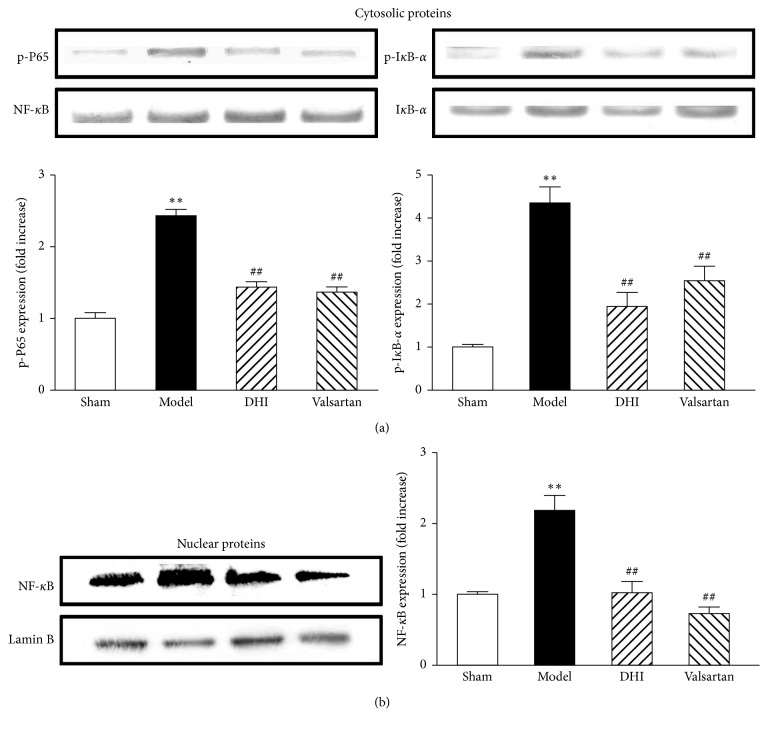
Effect of DHI on the activation of the NF-*κ*B pathway. (a) DHI inhibits phosphorylation of NF-*κ*B and I*κ*B-*α* in cardiomyocytes cytoplasm from infarct marginal zone. (b) DHI prevents the nucleation of NF-*κ*B in myocardial tissue from the infarct marginal zone. The level of p-NF*κ*B and p-I*κ*B-*α* in the cytoplasm was corrected by NF-*κ*B and I*κ*B-*α*, respectively. The expression of NF-*κ*B in the nucleus was corrected by Lamin B. Data are expressed as mean ± SD (*n* = 3). ^*∗∗*^*p* < 0.01 compared with the sham group; ^##^*p* < 0.01 compared with the model group.

The effect of DHI on NF-*κ*B pathway activation was examined in the cytoplasm and nucleus of cardiomyocytes from MI rats. The cytosolic phosphorylation of NF-*κ*B and I*κ*B-*α* was signiﬁcantly enhanced in the model group; however, this change was notably inhibited by DHI and valsartan treatment (Figure 6(a)). Additionally, the nuclear expression of NF-*κ*B was observed. Interestingly, MI not only increased the phosphorylation of NF-*κ*B in the cytoplasm but also markedly enhanced the expression of NF-*κ*B in the nuclear (*p* < 0.01, the model group compared with the sham group). However, Figure 6(b) showed that DHI and valsartan obviously inhibited the MI induced changes (*p* < 0.01, compared with the model group).

## 4. Discussion

A large number of evidence suggest that miRNAs play a critical role in diverse biological processes, such as embryogenesis, differentiation, carcinogenesis, immune system, inflammation, and viral infection, as well as involved in the physiological and pathophysiological processes such as cardiomyocytes apoptosis, angiogenesis, myocardial hypertrophy, ventricular remodeling, and heart failure in the cardiovascular system [6, 35–37]. Robust evidence has shown that regulation of miRNAs has an inseparable relationship with inflammation. More and more miRNA have been reported to be involved in the regulation of the immune system, such as the release of inflammatory mediators [35]. Firstly, we observed that DHI could obviously improve the LVEF and LVFS and increase the LVSP and ±*dp*/*dt* max in MI rats, which show that DHI can improve cardiac function and hemodynamics in rats after MI. In order to explore the mechanism of DHI improving cardiac function in MI rats, we used microRNA PCR array to predict and analyze its target.

In the present study, we obtained differentially expressed miRNA in myocardial tissue after MI using miRNA PCR array. Microarray screening showed that the expression of 20 inflammatory-related miRNA was modified after myocardial infarction, including 5 upregulated miRNAs and 15 downregulated miRNAs (Table 5). After the intervention of DHI, it was found that 25 miRNAs expression was upregulated (Table 6). We found that DHI can upregulate 8 miRNAs (let-7a-5p, let-7d-5p, let-7f-5p, miR-191a-5p, miR-142-5p, miR-26b-5p, miR-29b-3p, and miR-409a-3p), which are downregulated in myocardial infarction rats. The discovery of these miRNAs will provide a basis for future research.

miRNA-mRNA network analysis forecast results showed that the common target gene (miR-103-3p, miR-205, and miR-331-3p) IL-1*β* (miR-205, miR-142-5p, miR-129-5p, and miR-409a-3p), caspase-3, and (miR-130a-3p and miR-26b-5p) TNF-*α*. We tested the above target genes through PCR and found that DHI does affect the expression of TNF-*α*, IL-1*β,* and caspase-3 mRNA. This result is consistent with the previous reports that miRNAs can also act directly on the mRNA of cytokines to attenuate the inflammatory response and maintain the body immune balance [38].

Another evidence is that TNF-*α* binds to homologous receptors to raise and activate caspase-8 in animal models and in humans subjects, which in turn activates the main executioner of apoptosis and caspase-3 [39–41]. Moreover, miRNA-KEGG signal pathway analysis revealed that the differential miRNAs in the expression level regulating the molecular pathways mainly included the calcium signaling pathway, apoptosis, glycosylphosphatidylinositol-anchor biosynthesis, valine, leucine, and isoleucine degradation, PPAR signaling pathway, VEGF signaling pathway, natural killer cell-mediated cytotoxicity, purine metabolism, butanoate metabolism, amyotrophic lateral sclerosis, fatty acid metabolism, and long-term potentiation. DHI regulates these signaling pathways such as VEGF and apoptosis signaling pathway and has also been confirmed (data not shown). Therefore, our results suggest that DHI could protect the heart function of rats with MI by regulating miRNAs. This is a new mechanism by which DHI improves cardiac function in rats with myocardial infarction.

Another important evidence showed that IL-6 is the target for the Let-7 family of miRNAs, while Let-7 itself is also negatively regulated by TLR/NF-*κ*B [42, 43]. In the present study, DHI can significantly inhibit NF-*κ*B signaling pathway. It is well known that the transcription factor NF-*κ*B is closely involved in the inflammatory cascade, manifested by increased NF-*κ*B nuclear translocation, and promotes phosphorylation or degradation of I*κ*B-*α*. NF-*κ*B is a central transcriptional effector of inflammatory signaling which is involved in the regulation of cellular proliferation, differentiation, and apoptosis [31, 44]. Five subunits (RelA [p65], RelB, c-Rel, NF-*κ*B 1 [p50], and NF-*κ*B 2 [p52]) comprise the NF-*κ*B family [45]. It has been reported that p65 and p50 heterodimers are the main types of NF-*κ*B in cells and also the two most intensively studied subtypes of the NF-*κ*B family at the moment [46, 47]. NF-*κ*B activation and its subsequent nuclear translocation after MI trigger transcription of a large portfolio of genes including inflammatory cytokines (TNF-*α*, IL-1*β*, and IL-6), CXC, CC chemokines, and adhesion molecules [44]. Interestingly, our result agrees with the preservation of cardiomyocytes by DHI against inflammatory injury through the inhibition of the nuclear transcription of NF-*κ*B and I*κ*B-*α* phosphorylation. This indicates that DHI may have a pivotal role in alleviating the inﬂammatory injury caused by MI. These results provide evidence that the anti-inflammatory activity of DHI could be due to the regulation of the inflammation mediators via NF-*κ*B pathway at the MI process.

Although this study elucidates that DHI could resist myocardial infarction to some extent by regulating microRNAs, there are still many miRNAs that need further research. In particular, how DHI regulates miRNAs to improve cardiac function is a problem worthy of further study.

## 5. Conclusion

In summary, our study conﬁrmed that DHI ameliorates cardiac function and reduces myocardial infarct size via its anti-inflammatory effect through inhibiting NF-*κ*B pathway and regulating miRNA. In brief, DHI prevents the deterioration of inflammatory response by inhibiting nuclear translocation of NF-*κ*B and modulating inflammation-associated miRNA (such as miR-103-3p, miR-130a-3p miR-331-3p, miR-205, and miR-26b-5p), as well as regulating some target genes (TNF-*α*, IL-1*β*, and caspase-3) of the miRNA in the infarcted marginal zone. DHI inhibits myocardial cell apoptosis and promotes angiogenesis, thereby reducing infarct size and improving cardiac function. These findings may contribute to the understanding of molecular mechanisms involved in cardioprotection of DHI and provide novel insights into a future therapeutic strategy for early myocardial ischemia event.

## Figures and Tables

**Table 1:** Reverse transcription system and reaction conditions.

Array format: component	Volume	Reaction conditions
5x miScript HiSpec Buffer	4 *μ*l	
10x miScript Nucleics Mix	2 *μ*l	
RNase-free water	Variable	37°C 60 min
miScript Reverse Transcriptase Mix	2 *μ*l	95°C 5 min
Template RNA (added in step 3)	Variable	
Total volume	20 *μ*l	

**Table 2:** Reaction mix for the miRNA PCR array.

Array format: component	Volume (96-well)
2x QuantiTect SYBR Green PCR Master Mix	1375 *μ*l
10x miScript Primer assay	275 *μ*l
RNase-free water	1000 *μ*l
Template cDNA	100 *μ*l
Total volume	2750 *μ*l

*Note.* Ensure whether the cDNA was diluted 10 times (25 *μ*l per well).

**Table 3:** Cycling conditions for real-time PCR.

Step	Time	Temperature
PCR initial activation step	15 min	95°C
3-step cycling		
Denaturation	15 s	94 C
Annealing	15 s	55 C
Extension	30 s	70 C
Cycle number	40 cycles	

**Table 4:** Primers sequences used for real-time PCR.

mRNA (rat)	Sequence	
TNF-*α*	Forward	5′GAAGAGAACCTGGGAGTAGATAAGG3′
	Reverse	5′GTCGTAGCAAACCACCAAGC3′
IL-1*β*	Forward	5′TCGTTGCTTGTCTCTCCTTG3′
	Reverse	5′AAAAATGCCTCGTGCTGTCT3′
Bcl-2	Forward	5′ACAGCCAGGAGAAATCAAACA3′
	Reverse	5′GGTGGACAACATCGCTCTG3′
Caspase-3	Forward	5′AGTTTCGGCTTTCCAGTCAG3′
	Reverse	5′AGTTGGCATGGTAGCCCTTG3′
GAPDH	Forward	5′GGAGCAGTTTTGTGTGTGTGA3′
	Reverse	5′CTGGAAGATGGTGATGGGTT3′

**Table 5:** Differentially expression miRNAs genes in the model vs sham group (*n* = 3).

Position	Mature ID	Fold regulation
1	rno-let-7a-5p	−3.5319
2	rno-let-7d-5p	−2.0171
3	rno-let-7f-5p	−3.7884
4	rno-miR-126a-3p	−2.9236
5	rno-miR-128-3p	−2.0136
6	rno-miR-150-5p	−3.8158
7	rno-miR-191a-5p	−2.1912
8	rno-miR-142-5p	−2.4379
9	rno-miR-26b-5p	−3.9403
10	rno-miR-29b-3p	−2.7301
11	rno-miR-30b-5p	−2.3728
12	rno-miR-30c-5p	−2.3302
13	rno-miR-30e-5p	−2.243
14	rno-miR-26a-5p	−2.2767
15	rno-miR-409a-3p	−2.5957

1	rno-miR-146b-5p	4.1473
2	rno-miR-21-5p	2.6806
3	rno-miR-214-3p	5.1766
4	rno-miR-383-5p	2.1495
5	rno-miR-31a-5p	45.4912

**Table 6:** Differentially expression miRNAs genes in the DHI vs model group (*n* = 3).

Position	Mature ID	Fold regulation	Predicted target genes
1	rno-let-7a-5p	4.8760	IL-10, Ccl11
2	rno-let-7c-5p	2.2401	IL-10, Ccl11
3	rno-let-7d-5p	2.4142	IL-10, Ccl11
4	rno-let-7e-5p	2.2876	IL-10, Ccl11, Fadd
5	rno-let-7f-5p	2.8107	IL-10, Ccl11
6	rno-miR-103-3p	2.0075	Cxcl12, IL-1*β*, Fadd
7	rno-miR-129-5p	2.1133	Nox4, Vegf, Cxcl1, IL-7, Casp3
8	rno-miR-130a-3p	2.2469	Ccl2, Tnf, Cxcl12, Cxcl13
9	rno-miR-142-5p	3.1912	Tlr2, Cxcl12, Casp3, iNOS
10	rno-miR-143-3p	2.3556	Cxcl11, Cd40, Cxcl12, iNOS
11	rno-miR-15b-5p	2.2214	Cd40
12	rno-miR-16-5p	2.0139	Cd40
13	rno-miR-185-5p	2.5885	Ccl5, Crp, iNOS
14	rno-miR-186-5p	3.1615	Cebpb, Cxcl12, Cxcl13, Ccl13, Tlr4, Cxcl1, IL-10
15	rno-miR-191a-5p	2.1983	Cxcl11, Cxcl12, Cebpb
16	rno-miR-205	2.8986	Cxcl12, Casp3, IL-1*β*, iNOS
17	rno-miR-207	2.3392	Cxcl11, Cxcl12, Ccl21, serpinel, Crp, Cebpb
18	rno-miR-210-3p	2.3432	Cxcl12
19	rno-miR-26b-5p	2.0389	Vegf, tnf, Crp, Ccl3
20	rno-miR-296-3p	2.2710	Cxcl1, IL-7
21	rno-miR-299a-5p	2.0430	IL-10
22	rno-miR-29b-3p	3.9202	Nox, Ccl21, Ccl3
23	rno-miR-325-5p	2.3339	Ccl2, Cxcl12
24	rno-miR-331-3p	4.3646	Serpinel, IL-1*β*
25	rno-miR-409a-3p	3.3666	Casp3, IL-10

Positive values represent upregulation.

## Data Availability

The data used to support the findings of this study are available from the corresponding author upon request.
